# Supplementary material for: Phenolic degradation by catechol dioxygenases is associated with pathogenic fungi with a necrotrophic lifestyle in the Ceratocystidaceae
Source: G3 (Bethesda). 2022 Jan 13;12(3):jkac008. doi: 10.1093/g3journal/jkac008 (PMC8896014; doi:10.1093/g3journal/jkac008)
Supplement: jkac008_Supplementary_Data [file jkac008_supplementary_data.docx]

## Supplementary tables and figures


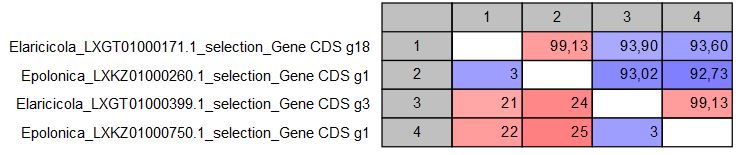


**Figure S1:** Gene duplication information for *E. polonica* and *E. laricicola* (amino acid percentage similarity shown in the upper right grouping of block and number of differences shown in the lower left grouping of blocks). Colours indicate level of similarity, with red most similar, and purple less similar.


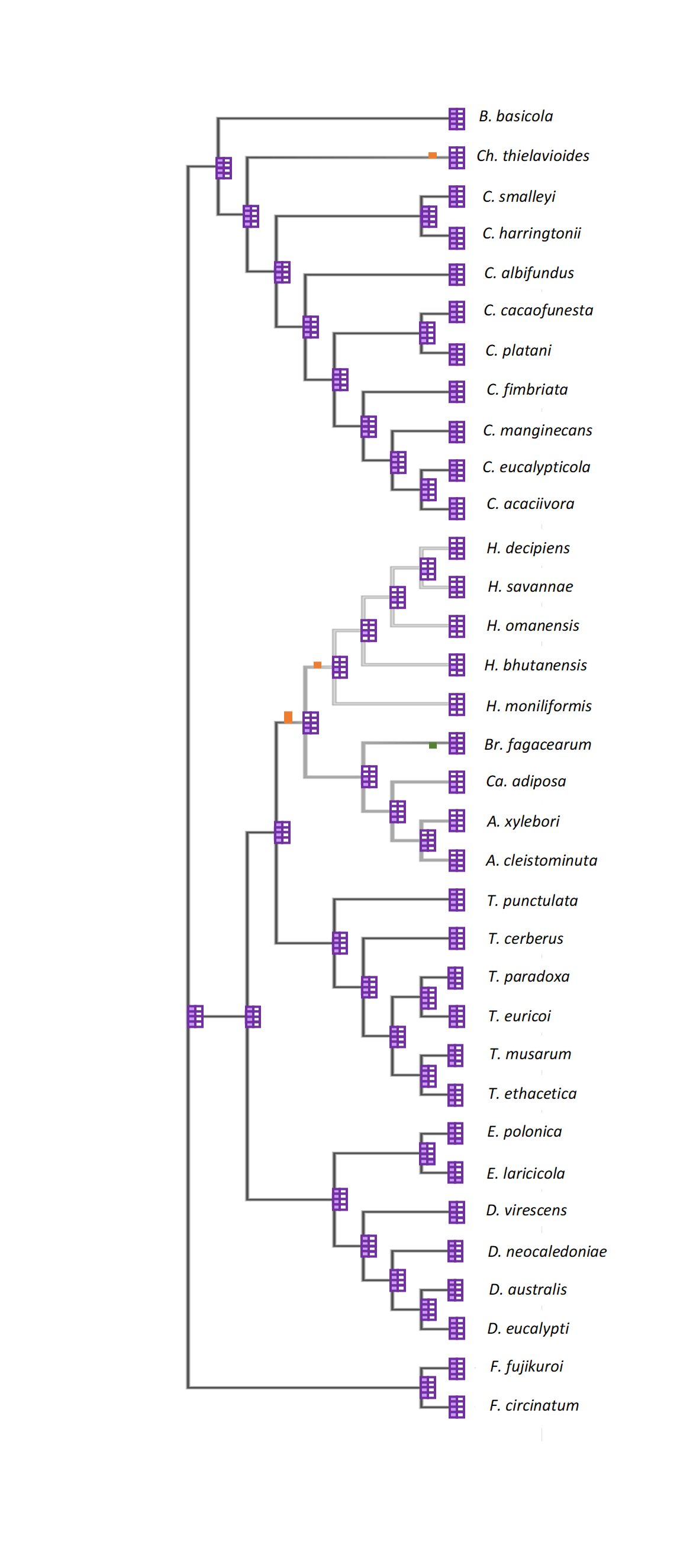


**Figure S2:** Ancestral history of *CDO 1-4* genes using a Wagner parsimony analysis in Count. Gene loss events are represented by orange blocks, and gene gains by green blocks. The *CDO* presence is evidenced through purple blocks at each node and at the terminal nodes.
